# Evaluating the efficiency and determinants of mass tourism in Spain: a tourist area perspective

**DOI:** 10.1007/s10258-022-00228-9

**Published:** 2022-12-12

**Authors:** Francisca J. Sánchez-Sánchez, Ana M. Sánchez-Sánchez

**Affiliations:** Universidad Pablo de Olavide, Ctra. Utrera Km 1. ES, 41013 Sevilla, España

**Keywords:** Mass tourism, Efficiency, Employment, DEA, Environmental factors, L83, R15, Z30

## Abstract

Tourism is one of the fastest-growing economic sectors. This has piqued increasing interest in the evaluation of the performance of the sector. This paper joins this line of research by providing a potential framework for measuring efficiency in the context of a country such as Spain, where sun-and-sand tourism, usually associated with mass tourism, predominates. Tourist areas located on the coast provide the units of reference. Data Envelopment Analysis (DEA) is applied to determine the efficiency score and a Tobit-type model is employed to analyse the factors that determine efficiency. The results show that the impact of mass tourism on labour efficiency is geographically unequal, with the most efficient of the tourist areas located on the peninsular archipelagos. The analysis of the contribution of each input to the efficiency score reveals the pre-eminent role of tourism infrastructure as a lure for sun-and-sand tourism.

## Introduction

Tourism is one of the fastest-growing economic sectors worldwide, and is fundamental for job creation and economic diversification (Sanchez and Sanchez 2018; Long 2011; Andereck and Nyaupane 2011). According to the World Tourism Organisation (UNWTO 2017), international tourism accounts for 7% of global exports of goods and services; its contribution to GDP globally is 10.4%, and it generates 1 in 10 jobs. Coastal destinations constitute the most appreciated environment, where sun-and-sand tourism has become a mass phenomenon where every year millions of people seek rest and recreation, preferably at the beach (Jedrzejczak 2004; Ley-Vega et al. 2007). The term *mass tourism* (Deprest 1997; Boyer 1999) was initially coined to describe a homogeneous, standardised, and rigid form of tourism, comparable to the mass production system introduced by Henry Ford (Poon 1993). Mass tourism is growing steadily thanks to the increased availability of free time and short-term package holidays. This type of tourism is large-scale, agglomerated, and highly seasonal. It is based on overnight stays in large hotel complexes, where tourists have consumerist habits, prefer sun-and-sand tourism, and visit large tourist destinations such as colonial cities or cities with cultural attractions located in large urban areas. Research on mass tourism that focuses on destinations in Mediterranean Europe shows that the factors most valued by sun-and-sand tourists are climate, beach quality, scenery, quality of facilities, recreational activities, safety, absence of litter, and attractiveness of the location (Jedrzejczak 2004; Williams 2011). However, other elements, such as culture and heritage, are becoming increasingly valued (Santana-Jiménez and Hernández 2011; Farmaki 2012).

The choice of Spain for the study allows us to contribute towards the literature with research regarding a leading country in the tourism sector, where the main engine of the country's economic growth is largely based on this activity (Balaguer and Cantavella-Jorda 2002). Competitiveness between tourism destinations is on the increase, and therefore institutions and managers have become aware of the need to improve their performance in order to remain competitive; to present a competitive advantage, hence efforts are therefore focused on the analysis of efficiency, which is key to management control and crucial in attaining better results. Efficiency analysis strives towards improvements in profitability and the optimal use of resources (Tavares 2002; Seiford 1997). The definition of efficiency states: "the ability to avoid waste, either by producing as much output as technology and input usage allow or by using as little input as required by technology and production function" (Fried et al. 2008, p. 5). This definition associates efficiency with the maximum output that can be achieved using given resources (inputs). This paper analyses labour efficiency in the tourism sector, understood as the minimum use of tourism resources (inputs) to achieve a given output or employment (output).

Assaf et al. (2017) call for an increase in the need to increase scientific production on efficiency that considers specific destinations. This research strives to extend previous studies by means of analysing territorial areas with accumulations of tourism of a specific type of activity. The Spanish National Statistics Institute (INE) defines a tourist area as "an area made up of a group of municipalities in which tourism is specifically located". One of the objectives of this research is to evaluate the efficiency of mass tourism in tourist areas along the Spanish coast, where sun-and-sand tourism is concentrated. Despite the importance and interest that the analysis of efficiency in these areas can present, the literature on efficiency in this type of territorial unit remains scarce (De Carlos Villamarín et al. 2016), although certain papers can be found that analyse efficiency both in terms of country (Lozano and Gutiérrez 2011) and of the region (Barros et al. 2011a, b; Huang et al. 2012; Brida et al. 2012; Benito-López et al. 2014; Sellers-Rubio and Casado-Díaz 2018).

There are various approaches to the measurement of the efficiency of the tourism sector (Wassenaar and Stafford 1991; Baker and Riley 1994; Ismail et al. 2002), one of which is Data Envelopment Analysis (DEA). In recent years, numerous papers have applied DEA methodology to analyse various aspects of the efficiency of tourism activity (Poldrugovac et al. 2016; Ohe and Peypoch 2016; Manasakis et al. 2013; Such-Devesa and Mendieta-Peñalver 2013; Assaf et al. 2012; Perrigot et al. 2009). Efficiency studies seldom include environmental variables, such as destination characteristics (Assaf et al. 2012, 2017; Assaf and Josiassen 2012), and there is therefore a need for research on these types of factors. This leads to the second objective of the paper, which seeks to determine the impact of various environmental factors on labour efficiency. In this context, variables related to the market and to the tourist destination that may influence efficiency are considered. For this purpose, the Analysis of Variance and a Tobit-type model are applied (Wang et al. 2006; Barros et al. 2011a, b; Huang et al. 2012; Parte-Esteban and Alberca-Olive 2015).

This paper is structured as follows. Section 2 studies the situation of the Spanish tourism sector. Section 3 presents the theoretical framework and defines the working hypotheses. Section 4 presents the analysis methodology and data sources. The results are presented in Section 5. Finally, in Section 6, the discussion and conclusions of the study are presented.

## Analysis of the spanish tourism sector

**Fig. 1:**
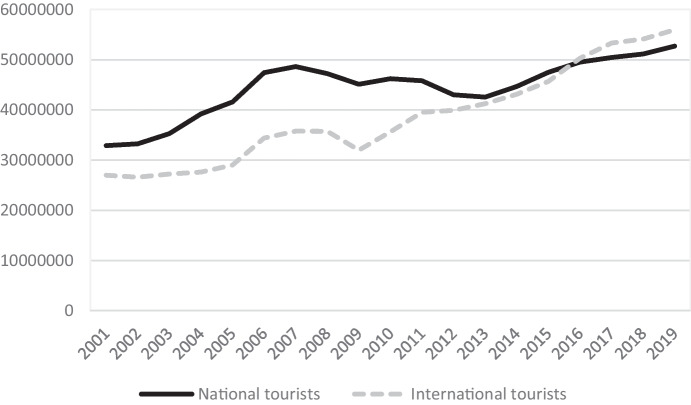
Evolution of the number of national and international tourists in Spain

The demand for leisure has expanded substantially in recent years due to the growth of the global economy. In Spain, holiday tourism has become a key activity. In 2019, the country ranked second in the world in number of tourist arrivals, only surpassed by France. Tourist demand, both national and international, is fundamental to the growth of the sector in Spain. In the last decade, international tourism in Spain has experienced a major increase, and has surpassed that of national demand (see Fig. 1). In 2019, 48.51% of tourists were national, while 51.49% were international, of which 48.27% were European, with 18 million English tourists, 11.15 million German tourists, and 11.14 million French tourists (INE 2019).

This significant flow of international tourist arrivals can be explained by the political problems experienced by some of Spain's direct competitors in sun-and-sand tourism, such as Egypt, Tunisia, and Greece. Moreover, several of these countries have entered a stage of tourism stagnation, which they are striving to solve by offering an experience to sun-and-sand tourism, with leisure and recreational by-products (Bonet 2003; Williams and Buswell 2003).

The profile of the international tourist visiting Spain is that of an individual between 25 and 44 years of age, salaried, with higher education and an average professional position, who lives with a partner, travels by air for leisure and recreational reasons, and tends to stay in hotel establishments. The level of satisfaction of international tourists with their visit to Spain is high (8.5 out of 10) and their loyalty to the destination is also high, according to the Institute of Tourism Studies (IET 2013).

**Fig. 2:**
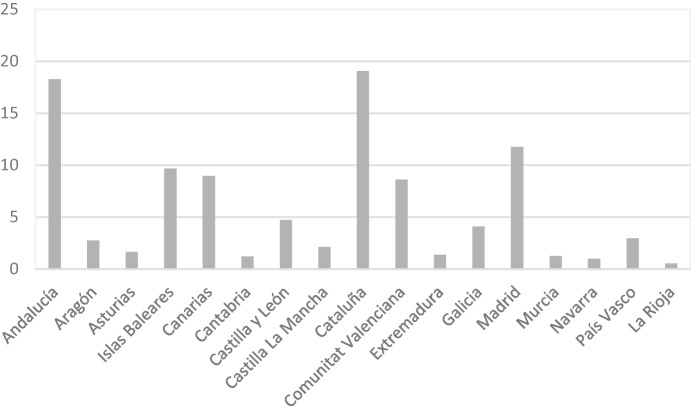
Tourists per destination (%)

In terms of the country's preferred destinations, the highest tourist occupancy was mainly in coastal areas, which underlines the visitor's preference for sun-and-sand tourism. Specifically, the regions of Catalonia, Andalusia, Valencia, Madrid, the Balearic Islands and the Canary Islands stand out, accounting for 76.36% of arrivals (Fig. 2).

**Fig. 3:**
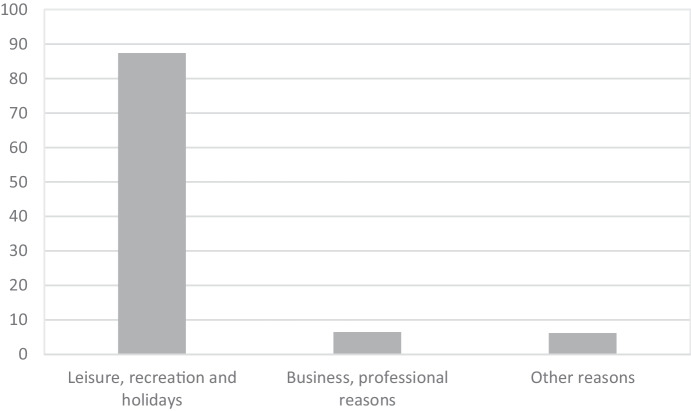
Visitor arrivals: reason for travel in 2019 (%)

These preferences for coastal destinations can be understood by looking at the main reasons for the trip. In 2019, leisure, recreation, and holidays, with 87.38%, constituted the main purpose of visits to Spain, while business, professional, and other reasons made up less than 13% (Fig. 3).

**Fig. 4:**
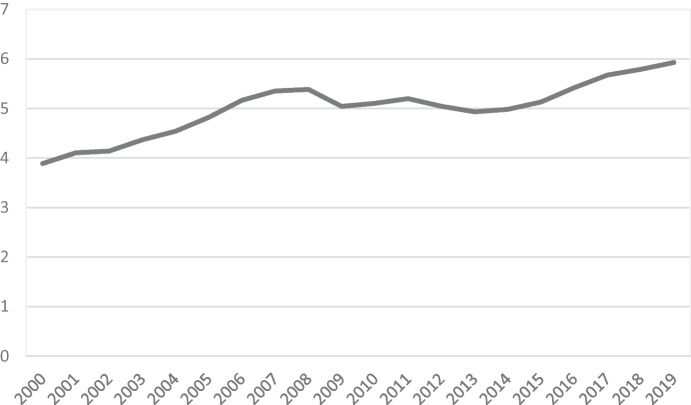
Evolution of employees in tourism

This major boom in the sector has contributed significantly to the generation of employment, since the number of people employed in tourism shows a steady and growing trend (Fig. 4). In 2019, the number of workers in the tourism sector affiliated to Social Security amounted to 2.72 million workers, which represents approximately 12.9% of the total number of employees in the whole country (INE 2019). The employment potential of the sector is enormous, although certain groups, such as women, young people, and older adults, have traditionally been marginalised in the labour market (Pérez 2010); however, these jobs do have particular characteristics, especially regarding their seasonal nature and insufficient professionalisation (Pou 2012).

**Fig. 5:**
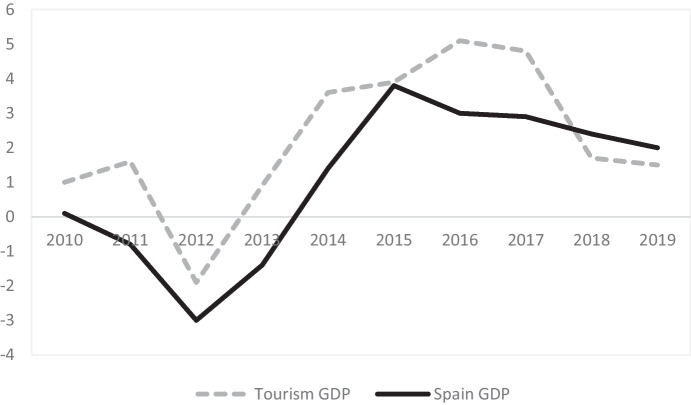
Evolution of the year-on-year rate of change of tourism GDP and the year-on-year rate of change of the GDP of the Spanish economy

In 2019, tourism contributed a total of 154,487 million euros to Spain's Gross Domestic Product (GDP), which represents 12.4% of its GDP of that year. An example of this importance can be seen in Fig. 5, which shows a comparison of the evolution of tourism GDP compared to national GDP. The tourism sector generates highly significant income for the country, with an increase in tourism expenditure of more than 21,000 million euros in the last four years. The year 2019 ended with the highest volume of tourism activity in Spain to date: more than 140,000 million euros (INE 2019).

## Research framework and working hypotheses

The evaluation of efficiency is fundamental for the tourism sector: it helps managers to make decisions by estimating performance, and provides companies with a competitive advantage. Therefore, institutions are also concerned with the analysis of the performance of tourism activities, given their enormous economic and social impact (Benito-López et al. 2014; Detotto et al. 2014; Solana-Ibáñez et al. 2016; Sellers-Rubio and Casado-Díaz 2018; Casado-Díaz and Sellers-Rubio 2020).

In studies into tourism efficiency, the sample, the methodology used, and the inputs and outputs employed are all fundamental (Assaf and Tsionas 2019; Assaf and Josiassen 2012). Regarding the sample studied, these papers propose two lines of research: the first examines samples of hotels in selected tourist destinations, while the second evaluates hotel brand efficiency. In reference to the first line of research, the literature mainly analyses destinations located in Asia (Yu et al. 2019; Liu et al. 2017; Yi and Liang 2015; Huang et al. 2012; Bi et al. 2011; Wang et al. 2007), in the United States (Gu, 2004; Brown and Ragsdale, 2002), and in Europe (Amado et al. 2017; Oliveira et al. 2013a; Brida et al. 2012; Barros et al. 2011a). In the case of Spain, there is the work of Deng et al. (2019), Arbeló et al. (2017), Arbeló-Perez et al. (2017), Solana-Ibáñez et al. (2016), Fernández and Becerra (2015), Parte-Esteban and Alberca-Oliver (2015), Benito-López et al. (2014), and De Jorge and Suárez (2014).

As for the methodology employed to assess efficiency, initially non-frontier-type models were applied (Wassenaar and Stafford, 1991; Baker and Riley, 1994), although nowadays, models based on the frontier concept are more widely used. The latter approach uses two different study perspectives: Data Envelopment Analysis (DEA) and the parametric stochastic frontier method. The DEA methodology (Charnes et al. 1978) is a non-parametric linear programming method (see, for example, the works of Yuan and Liu 2020; Kularatne et al. 2019; Soysal-Kur 2017; Yi and Lian 2015). In recent years, numerous studies have applied DEA for the study of tourism efficiency in various contexts: in hotel studies (Higuerey et al. 2020; Lado-Sestayo and Fernández-Castro 2019; Karakitsiou et al. 2018; Solana-Ibáñez et al. 2016; Ohe and Peypoc, 2016; Manasakis et al. 2013; Assaf et al. 2012), in travel agencies (Dragan et al. 2018; Ramírez-Hurtado and Contrera, 2017; ZhiYi 2015; Fuente 2011; Barros and Diek 2007), and in airlines (Shirazi and Mohammad, 2019; Yu et al. 2019; Sakthidharan and Sivarama, 2018; Sing 2011; Barbot et al. 2008). Parametric stochastic frontier methods (Aigner et al. 1977; Meeusen and van den Broec 1977) are based on econometric models. This methodology is used in the analysis of the efficiency of the tourism sector (Deng et al. 2019; Arbeló et al. 2017; Arbeló-Pérez et al. 2017; Bernini and Guizzardi 2016, 2010; Guetat et al. 2015; Oliveira et al. 2013b; Kim 2011).

The DEA methodology enjoys several advantages over stochastic frontier methods. Multiple outputs can be considered, while in the stochastic frontier methodology only one output can be used. Furthermore, DEA is a non-parametric technique, and therefore it does not need to consider a functional form in order to establish the relationship between inputs and outputs, whereas in stochastic frontier techniques this form is necessary.

The selection of inputs and outputs has generated debate in the literature (Perrigot et al. 2009). In practice, the choice of inputs and outputs is made according to data availability and to experience in formulating and implementing models (Hwang and Chang 2003). The inputs and outputs commonly used in research on tourism efficiency are classified into physical variables, of which the most common are the number of employees and the number of available job vacancies (Oukil et al. 2016; Assaf et al. 2015b; Chen 2009; Hwang and Chang 2003). Financial variables, such as employee wages, operating costs, profits, revenues and sales, have also been considered (Detotto et al. 2014; De Jorge and Suárez 2014; Barros et al. 2011a; Wang et al. 2007).

In Spain, studies on tourism efficiency are both scarce and relatively recent (Benito-López et al. 2014; De Jorge and Suárez 2014; Parte-Esteban and Alberca-Oliver 2015; Fernández and Becerra 2015; Solana-Ibáñez et al. 2016; Arbeló et al. 2017; Deng et al. 2019; Sánchez-Sánchez and Sánchez-Sánchez 2021). The majority of these studies carry out analysis at the regional level; however, there is a lack of research that assumes the geographical perspective of tourist areas. In this context, the study of the impact of mass tourism (predominant in Spain) on the efficiency of the labour market in terms of tourist areas can make makes a significant contribution to the existing literature. This leads us to formulate the following research hypothesis:

Hypothesis 1: *Mass tourism is an activity that has a relative impact on labour efficiency in coastal tourist areas*.

Once the impact of mass tourism on tourist efficiency has been studied, the work is complemented by another research objective, which analyses the influence of certain environmental factors of the tourist destination on efficiency. Lovell (1993) indicates that “the identification of the factors that explain differences in efficiency is essential for improving the results of firms although, unfortunately, economic theory does not supply a theoretical model of the determinants of efficiency”. There are numerous factors influencing efficiency that are not controlled for (Coelli et al. 1998). For this reason, the study of models and determinants of tourism destinations has recently become of great interest (see, for example, Mazanec et al. 2007; Tsai et al. 2009; Crouch 2011; Assaf and Josiassen 2012; Benito-López et al. 2014; Marco-Lajara et al. 2014). Existing work explains efficiency by identifying its determining factors (Assaf and Josiassen 2012; Assaf et al. 2012; De Jorge and Suárez 2014; Parte-Esteban and Alberca-Oliver 2015; Sellers-Rubio and Casado-Díaz 2018). These determinants consider several factors: the tourist destination (Sellers-Rubio and Casado-Díaz 2018; Yang and Cai 2016; Assaf et al. 2015a); infrastructure, services, interest, and image projected by the destination (Assaf and Josiassen 2012); tourism management factors (Hwang and Chang 2016; De Jorge and Suárez 2014; Xiao et al. 2012); and environmental factors (Chen et al. 2018; Sellers-Rubio and Casado-Díaz 2018; Solana-Ibáñez et al. 2016; Assaf et al. 2012; Shieh 2012; Wang et al. 2006). For all these reasons, and in order to complete the efficiency study, it is interesting to analyse the variables that influence efficiency, especially concerning the impact of environmental variables. For the analysis of these environmental factors, four variables are considered: (1) Length of stay; (2) Number of international tourists; (3) Hotels with quality distinction; and (4) Location of the tourist destination. The first two variables are related to the market, while the last two variables are associated with the tourist destination.

The *length of stay* variable is intended to measure a crucial temporal aspect for the tourism industry, given that tourists with longer stays generate a stronger economic, social, and environmental impact than those with shorter stays (Barros and Machado 2010; Parte-Esteban and Alberca-Oliver 2015; Sellers-Rubio and Casado-Díaz 2018). This variable captures the tourist's perception of the tourist destination: the longer they stay at their destination, the greater its perceived attractiveness (Botti et al. 2008).

The *number of international tourists* exerts a strong impact on tourism efficiency (Hwang and Chang 2003; Chen 2010; Huang et al. 2012; Ben Aissa and Goaied 2016; Assaf et al. 2017; Sellers-Rubio and Casado-Díaz 2018), since international tourists stay longer and spend more (Rosenbaum and Spears 2006).

The quality control variable can present a competitive advantage for companies in the sector (Akbaba 2006; Chen 2013). In the hotel industry, quality is usually measured through the hotel category, that is, its number of stars. However, this star rating has been questioned as an indicator of quality (Núñez-Serrano et al. 2014; López-Fernández and Serrano-Bedia 2004), and it has even been shown that it is not an important indicator in the measurement of efficiency (Oliveira et al. 2013a). Another way to measure hotel quality is through quality assurance programmes (Brown and Ragsdale 2002; Claver et al. 2006). Certain research positively relates efficiency to the number of quality certificates (Claver-Cortés et al. 2007; Costa 2004), while other studies establish a negative relationship (Sellers-Rubio and Casado-Díaz 2018; Fernández and Becerra 2015).

The location of the tourist destination can determine the occupancy rate of hotels and their profitability (Lado-Sestayo et al. 2016). On the other hand, the location of the destination has also been a factor commonly considered in factor analysis, whereby it is found that tourist areas located on the coast tend to show higher efficiency than other areas (Barros et al. 2011a; Benito-López et al. 2014; Solana-Ibáñez et al. 2016; Sellers-Rubio and Casado-Díaz 2018), although certain studies do find a negative relationship (Parte-Esteban and Alberca-Oliver 2015).

The analysis of environmental factors that determine efficiency leads to the following working hypotheses:

Hypothesis 2: *Tourist destinations located on peninsular archipelagos have higher labour efficiency than other tourist areas*.

Hypothesis 3: *The length of the tourist stay has a direct and positive relationship with the labour efficiency of tourist areas*.

Hypothesis 4: *The number of international tourists has a direct and positive relationship with the labour efficiency of tourist areas*.

Hypothesis 5: *The quality of hotels has a direct and positive relationship with the labour efficiency of tourist areas*.

Table 1 provides a summary of the various papers that apply DEA to efficiency measurement and that propose a second stage of research for the analysis of the relationship between efficiency and its possible determining factors.

**Table 1:** Survey of the literature on DEA models in tourism and their determining factors

Research	Period	Model	Units studied	Inputs	Outputs	Second-stage explanatory variables
Barros (2005)	Annual data 1999–2001	DEA Malmquist with second-stage Tobit regression	42 Hotels in Portugal	Number of full-time employees, operating costs, cost of labour, book value of property	Number of guests, number of nights occupied, sales	Locational competitive position, ratio of the workers of the hotel versus the total number of Enatur's workers, distance to the airport, number of rooms, value of yearly investment, city or remote
Wang et al. (2006)		DEA with second-stage Tobit regression	49 hotels in Taiwan	Number of full-time employees, guest rooms, total floor space of catering división	Room revenue, food and beverage revenue, other revenues (operating revenues from lease of store spaces, laundry, swimming pool, sports facilities, barbers’, beauty salons, and bookstores)	Market conditions, management style, hotel size
Barros and Dieke (2008)	Annual data 2000–2006	DEA Malmquist with second-stage bootstrapped Tobit model	12 Hotels in Luanda (Angola)	Investment expenditure, total cost	Revenue per available room	Trend, square trend, market share, hotels belonging to an economic group, hotel groups with an international expansion strategy
Barros et al. (2011a)	Annual data 1998–2005	DEA with second-stage Tobit regression	15 Hotels in Portugal	Number of full-time workers, book value of property, operating costs	Sales, number of tourists	Trend, square trend, quoted hotel groups, companies involved in mergers and acquisitions, hotels belonging to an economic group, hotel groups with an international expansion strategy
Barros et al. (2011b)	Annual data 2003–2007	DEA with second-stage Tobit regression	Hotels in 22 French regions	Accommodation capacity, tourist arrivals	Number of nights	Monuments, museums, theme parks, beaches, ski resorts, and Natural Parks
Shieh (2012)	Annual data 1997–2006	DEA with second-stage Tobit regression	547 Hotels in Taiwan	Number of guest rooms, number of employees, total floor space of catering división	Total revenue of food and beverages, total revenue of rooms, other revenues (operating revenues from lease of store spaces, laundry, swimming pool, ball courts, barbers’, beauty salons, and bookstores)	Type of location, type of operation, distance to nearest international airport, the occupancy rate, level of green areas
Huang et al. (2012)	Annual data 2001–2006	DEA with second-stageTobit regression	Hotels in 31 regions of China	Number of full time employees, number of tourists, total fixed assets	Total income, average occupancy rate	Historical average of the technical efficiency score, richness of tourism resource, international tourism attractiveness, education, payment levels of employees, market competition, regional trade openness, a time (2003) dummy variable
Benito-López et al. (2014)	Annual data 2002–2010	DEA with second-stage bootstrapped Tobit model	Hotels in 17 regions of Spain	Number of accommodation places, number of tourists	Number of beds	Presence of ski resorts, number of restaurants, number of cultural properties, number of museums, natural surface, number of golf clubs, number of retailers, percentage of meeting attendance, coastal destination
Oukil et al. (2016)	-	DEA with second-stage bootstrapped Tobit model	58 Hotels in Oman	Number of beds, salary of employees	Number of nights, number of guests, annual revenue, occupancy rate	Star rating, size, nature, ownership, activities, culture
Detotto et al. (2014)	Annual data 2000–2006	Window-DEA, double-bootstrapped, pooled-truncated regression	21 regions in Italy	Gross fixed investment, labour costs	Value added, sales revenue	Share of high-quality hotels per región, rate of utilisation of beds, annual coefficient of variation of the net rate of utilisation, art city
Parte-Esteban and Alberca-Oliver (2015)	Annual data 2001–2010	DEA with second-stage Tobit regression	1385 hotels in Spain	Book value of property, number of full-time workers, operational costs	Sales	Bed capacity, hotel occupancy rate, number of arrivals, number of visitors staying overnight, gross domestic product, dummy variable of location in coast, return on assets, leverage, size, prices earnings ratio, dummy variable for hotel situated in a region above the national average in terms of tourist flow
Solana-Ibáñez et al. (2016)	Annual data 2005–2013	DEA Malmquist with second-stage bootstrapped Tobit model	Hotels in 17 regions of Spain	Number of bed nights	Accommodation capacity, number of beds, tourist arrivals	Tourist attractions and services, coastal destination, number of cultural assets, number of museums and art collections, percentage of attendance at meetings, number of federated golf clubs, number of restaurants, number of retailers, number of hotels, number of retailers
Sellers-Rubio and Casado-Díaz (2018)	Annual data 2008–2016	DEA with second-stage Tobit regression	Hotels in 17 regions of Spain	Number of hotels, number of beds, number of employees	Average daily expenditure, revenue per room, average occupancy rate	Length of stay, number of international tourists arriving in Spain, sun-and-sand tourism product, number of hotels distinguished with a quality label
Kularatne et al. (2019)	Annual data 2010–2014	DEA Malmquist with second-stage bootstrapped Tobit model	24 Hotels in Sri Lanka	Number of employees, number of rooms, book value of assets	Room revenue, other revenues	Number of Years of the hotel, hotel size, star rating of the hotel, type of hotel, eco-friendly practices in energy-saving, eco-friendly practices in water-saving, eco-friendly practices in waste management

Source: Authors’ own

## Methodology and sources

The data employed herein is taken from official statistics published by the National Statistics Institute (INE). Specifically, the data comes from the Hotel Occupancy Survey for the year 2019. This year has been selected because it is the one prior to the beginning of the COVID-19 health crisis, since the aim of the study is to evaluate the efficiency of mass tourism in tourist areas of the Spanish coast, in a context of "normality", without external disturbances. For this reason, a more recent time period is not considered, given that the special situation produced by the pandemic would not reflect the reality of the sector. The Hotel Occupancy Survey survey is carried out on a monthly basis, and its population scope includes all hotel establishments in Spain. This Survey offers information on the demand side, where data is provided on travellers, overnight stays and average stay distributed by country of residence for non-resident travellers in Spain and the category of the establishments they occupy, or by Autonomous Community of origin in the case of Spanish travellers. On the supply side, the estimated number of establishments open, estimated bedplaces, occupancy rates and information on employment in the sector, according to the category of establishment, are provided.Official statistics published by organisations such as Exceltur (Business Tourism Assessment 2019 and Outlook for 2020) and the Bank of Spain (National Accounts Statistics of the Spanish Economy) have also been used. It also uses data published by INE on the Tourism Satellite Account, which provides complete information on the economic relevance of tourism in Spain through macroeconomic indicators such as the contribution of tourism to GDP, employment, production and demand.

In order to measure mass tourism, this work studies the sun-and-sand tourism on the Spanish coast. The coastal tourist areas provide the unit of analysis of the study, and hence the choice of the study variables is conditioned by the availability of information in the databases. The INE defines a tourist area as "an area made up of a set of municipalities in which tourist inflow is specifically located". This study considers the 31 tourist areas of the Spanish coastline.

For the selection of variables, it has been taken into account that tourism fundamentally manifests itself in terms of hotel occupancy (Ivanov and Zhechev 2012; Assaf and Barros 2011; Chen 2010, 2011) and as employment or job performance (Karatepe 2012; Li et al. 2012). Many of the variables employed in the study are frequently used in the literature to study tourism efficiency (see, for example, the works of Deng et al. 2019; Sellers-Rubio and Casado-Díaz 2018; Soysal-Kurt 2017; Hadad et al. 2012; Huang et al. 2012; Lozano and Gutiérrez 2011; Hwang and Chang 2003).

The data matrix for the efficiency analysis consists of 5 variables collected for each of the 31 coastal tourism areas. For the data envelopment analysis (DEA), one output, in the form of the number of employees, is considered (Cvetkosa and Barišić 2017; Sánchez-Sánchez and Sánchez-Sánchez 2021), together with four inputs: number of tourists (Barros et al. 2011b; Huang et al. 2012; Benito-López et al. 2014); number of overnight stays (Johns et al. 1997; Solana-Ibáñez et al. 2016); number of hotels (Sellers-Rubio and Casado-Díaz 2018; Sánchez-Sánchez and Sánchez-Sánchez 2021); and number of room vacancies (Hwang and Chang 2003; Barros et al. 2011b; Benito-López et al. 2014; Oukil et al. 2016; Sellers-Rubio and Casado-Díaz 2018).

Four variables will be employed to study the impact of environmental variables on tourism efficiency: length of stay (Parte-Esteban and Alberca-Oliver 2015; Sellers-Rubio and Casado-Díaz 2018); number of international tourists (Ben Aissa and Goaied 2016; Assaf et al. 2017; Sellers-Rubio and Casado-Díaz 2018); hotels with quality distinction (Fernández and Becerra 2015; Poldrugovac et al. 2016; Oukil et al. 2016; Sellers-Rubio and Casado-Díaz 2018; Kularatne et al. 2019); and tourist destination location (Solana-Ibáñez et al. 2016; Parte-Esteben and Alberca-Oliver 2015; Oliveira et al. 2013a; Bernini and Guizzardi 2010). The description of the variables considered, together with several associated statistical measures, are listed in Table 2.

**Table 2:** Description of variables

Variables	Description	Units	Mean	Standard deviation
Variables DEA	Employees: workers in the tourism sector in the tourist areas studied	People	5,800.000	6,320.257
	Tourists: people on a tourist trip	People	1,936,799.060	1,803,178.774
	Overnight stays: each night a traveller stays in a hotel	People	9,126,303.260	9,841,352.190
	Hotels: number of hotels used for tourism activities	Number	182.900	144.202
	Bed capacity: total number of beds available in a hotel	Number	35,616.390	34,495.724
Tobit Variables	Quality: number of hotels recognised with a quality distinction, defined as Q for tourism quality	Number	63.580	50.119
	Stay: number of days a tourist spends at a given destination	Days	4.020	1.868
	Foreigners: number of international tourists	People	8,777,595.250	11,144,361.061
	Island: Dummy variable that takes the value 1 if the tourist area is located on an island and 0 otherwise	Number	-	-

Source: Authors' calculations

Note that the variables considered in the analysis of environmental factors were not selected as inputs in the DEA model so as not to reduce the explanatory power of the DEA model.

The DEA methodology must comply with the condition that all inputs must be positively related to at least one output (Sigala et al. 2004; Chiang 2006; Perrigot et al. 2009). The isotonicity test enables this rule to be verified by testing the statistical significance of the correlation between the variables considered. Table 3 shows Pearson’s correlation coefficients and the p-values (p) between inputs and output. The inputs studied are statistically significant, and are positively correlated with the output, and hence the selection of variables is correct and all the variables selected comply with the rule for the application of the DEA methodology. Furthermore, it can be observed that the inputs are positively related to each other, which indicates that the coastal tourist areas with the most tourists are those with the most overnight stays, the most hotels, and the most hotel beds.

**Table 3:** Pearson's correlation coefficients and p-values

	Employees	Places	Hotels	Overnight stays	Tourists
Employees	1	0.977^**^	0.560^**^	0.983^**^	0.878^**^
		(0.000)	(0.001)	(0.000)	(0.000)
Places		1	0.647^**^ (0.000)	0.995^**^ (0.000)	0.950^**^ (0.000)
Hotels			1	0.596^**^	0.805^**^
				(0.000)	(0.000)
Overnight stays				1	0.929^**^
					(0.000)
Tourists					1

**p < 0.01

Source: Authors' calculations

### Data Envelopment Analysis (DEA)

Data Envelopment Analysis (DEA) is a non-parametric technique employed to compare the efficiency of a set of organisations (DMU) based on data containing information on certain variables. The variables are classified as inputs or outputs according to the production process, that is, inputs are the resources that influence the production of outputs.

The DEA methodology assigns an efficiency score to each DMU. If the efficiency score for a given DMU is equal to 1, then that DMU is efficient; if a DMU scores below 1, then it is inefficient.

Charnes et al. (1978) defined the standard input-oriented CCR DEA model. In this model, U = {1,2,…,u} is considered to be a set of independent DMUs, each consuming a set of different inputs, I = {1,2,…,n}, in amounts ${x}_{ij},$ to create a set of different outputs, O = {1,2,…,m}, in amounts ${y}_{kj}$. The efficiency value of a DMU, ${j}_{0} \epsilon U$, is calculated through the following linear programming problem:

$$\begin{array}{ccc}E\left(j_0\right)=&\min\ \theta_{j_0}&\\&s.t.&\sum\limits_{j\in U}\lambda_jx_{ij}\leq\theta_{j_0}x_{{ij}_0} \; for\;all\;i\;\in I\\&&\sum\limits_{j\in U}\lambda_jy_{kj}\geq y_{{kj}_0} \; for\;all\;k\in O\end{array}$$

$$\begin{array}{ccc}& & \\ & {\lambda }_{j}\ge 0 & for\ all\ j\ \in U\setminus \{{j}_{0}\}\\ & {\theta }_{{j}_{0}}\, free.& \end{array}$$

According to the efficiency scores obtained, a ranking of inefficient DMUs can be defined. The efficient DMUs, however, have the same efficiency value, and therefore they cannot be ranked. In DEA, there are various ways to obtain an additional ranking of efficient DMUs. Two of the most commonly used methods are the Global Leader (Oral and Yolalan 1990) and the super-efficiency score (Andersen and Petersen 1993). The term Global Leader highlights the unit with the best overall performance. The efficient unit that appears most frequently in the reference sets of inefficient units is classified as the Global Leader. The super-efficient score method excludes the DMU, for which the efficiency value is calculated, from the initial set of DMUs. This method can reach values larger than 1 and can be applied to rank all DMUs. The super-efficiency method has the advantage over other methods in that it is applied to sort only the efficient DMUs, since the super-efficiency values of the inefficient DMUs are the same as their efficiency values. This is the method selected in the study to establish the ranking of efficient units.

The super-efficiency value of each DMU, ${j}_{0} \epsilon U$, for the standard input-oriented CCR DEA model is obtained as follows:

$$\begin{array}{ccc}{E}^{super}\left({j}_{0}\right) =& \mathrm{min\ }{\theta }_{{j}_{0}} & \\ & s.\mathrm{t}.& \sum\limits_{j \in U\setminus \{{j}_{0}\}}{\lambda }_{j}{x}_{ij}\le {\theta }_{{j}_{0}}{x}_{{ij}_{0 }} \; for\ all\ i \in I\\ & & \sum\limits_{j \in U\setminus \{{j}_{0}\}}{\lambda }_{j}{y}_{kj}\ge {y}_{{kj}_{0 }} \; for\ all\ k \in O\end{array}$$

$$\begin{array}{ccc}& & \\ & {\lambda }_{j}\ge 0 & for\ all\ j\ \in U\setminus \{{j}_{0}\}\\ & {\theta }_{{j}_{0}} \; free.& \end{array}$$

### Analysis of variance and the tobit model

In order to examine whether work efficiency is determined by various environmental variables, Analysis of Variance and a Tobit-type specification are applied.

The analysis of variance, also known as ANOVA model, basically allows comparing the mean values of the dependent variable in J populations where the factor levels are different, in order to determine if there are significant differences according to these levels or if, on the contrary, the response in each population is independent of the factor levels. The null hypothesis tested in the one-factor ANOVA is that the population means are equal. In our case, two groups of tourist areas are distinguished, those with efficiency values above the sample mean and those with scores below the mean efficiency score. This analysis will allow us to check whether the differences in the mean scores of the inputs studied in the two groups are random. The values of the test statistic will show the degree of importance of the inputs in explaining the labour efficiency of the tourist areas (the higher the test statistic, the greater the importance of the input). The analysis of variance methodology will also be applied to study the possible relationship between the input efficiency measure and the location of the tourist area. For this purpose, two groups of tourist areas will be considered, depending on whether the area is located on the mainland or in one of the archipelagos, analysing whether or not there are significant differences in the mean values obtained in the efficiency scores of these groups.

The Tobit model or limited dependent variable regression model is an alternative model to the logit and probit models. In this model, the sample is censored, i.e. the regression can be left-censored or right-censored (or even both) or truncated; so you can have a censored sample model or truncated,^1^ in which the dependent variable assumes values between 0 and 1. The Tobit model is a restricted dependent variable model (Greene 2004), and the explained variables can only be observed under restricted conditions.The basic form of the Tobit model is shown in the following specification:

$${E}_{j}^{*}=\alpha +{Z}_{j}\delta +{\varepsilon }_{j}$$, $${\varepsilon }_{j}\sim N(0,{\sigma }_{\varepsilon }^{2})$$ j = 1, …, n,

$${E}_{j}=\left\{\begin{array}{c} {{E}_{j}^{*}, E}_{j}^{*}\in \left[\mathrm{0,1}\right]\\ 0, {E}_{j}^{*}<0, or\ {E}_{j}^{*}>1\end{array}\right.$$

where ${E}_{j}^{*}$ is the dependent variable vector, that is, the efficiency score of the DMU (j), i.e. the labour efficiency score of tourist area j; α is the model constant, ε_j_ is a white-noise variable, Z_j_ is a vector of environmental variables that are expected to be related to the efficiency score, and ${E}_{j}$ is the observed value, i.e. the restricted value of the efficiency of the tourism sector in Spain. When ${E}_{j}^{*}\in \left[\mathrm{0,1}\right]$, ${E}_{j}$ equals ${E}_{j}^{*}$, which is the efficiency value that can be observed, but when ${E}_{j}^{*}\notin \left[\mathrm{0,1}\right]$, can’t be observed.

In our case, the empirical Tobit model would be as follows:

${E}_{j}={\alpha }_{0}+{\delta }_{1}{Foreigners}_{j}+{\delta }_{2}{Length\ of\ stay}_{j}+{\delta }_{3}{Quality}_{j}+ {\varepsilon }_{j}$where E_j_ is the labour efficiency variable for Spanish tourist areas, adopting the efficiency value measured by DEA; α_0_ is the constant term; ${\delta }_{1}$, ${\delta }_{2}$, ${\delta }_{3}$ are the parameters to be estimated and ε_j_ is a white-noise variable. *Foreigners* is the number of foreign tourists. Normally, high values of the variable coincide with tourist areas that are characterised by a good performance of tourism employment, so a negative sign is expected. In other words, high levels of foreign tourists are associated with low levels of (in)efficiency. *Length of stay* is the duration (in days) of the holiday stay, high values of the variable are expected to pertain to tourist areas with high tourism performance values. Therefore, the relationship between E and Length of stays is expected to be negative. Quality is the number of hotels recognised with a quality distinction. Normally, tourist areas with a high quality of tourist facilities have good employment efficiency values. Hence, a negative sign is expected.

## Results

The analysis of labour market efficiency caused by mass tourism is presented by distinguishing two cases: firstly, the efficiency ranking by tourist areas is presented; and secondly, the analysis of environmental factors is realised.

### Labour efficiency per tourist area

DEA is applied to identify labour-efficient tourism zones. Table 4 presents the ranking of labour-efficient tourist areas, and shows the efficiency score of these areas in decreasing order. The results indicate that there are only 3 tourism areas that reach labour efficiency (Isla de la Gomera, Sur de Tenerife, and Costa da Morte).

**Table 4:** Ranking and efficiency scores per tourist area

	Tourist area	Efficiency score		Tourist area	Efficiency score
1	Isla de la Gomera	1.251	17	Costa Guipuzkoa	0.736
2	Sur de Tenerife	1.133	18	Rías Baixas	0.735
3	Costa da Morte	1.048	19	Costa Verde	0.711
4	Isla de Fuerteventura	0.973	20	Costa de la Luz de Cádiz	0.701
5	Isla de Tenerife	0.965	21	Isla de Menorca	0.689
6	Costa a Mariña Lucense	0.963	22	Costa Valencia	0.640
7	Sur de Gran Canaria	0.922	23	Rías Altas	0.634
8	Isla de Gran Canaria	0.862	24	Costa Cálida	0.631
9	Isla de Lanzarote	0.861	25	Costa Blanca	0.616
10	Costa Bizkaia	0.790	26	Costa de Almería	0.602
11	Costa del Sol	0.772	27	Costa de Castellón	0.599
12	Palma—Calvía	0.764	28	Costa Brava	0.555
13	Isla de la Palma	0.760	29	Costa Tropical	0.553
14	Islas de Ibiza-Formentera	0.755	30	Costa Barcelona	0.550
15	Costa de La Luz de Huelva	0.749	31	Costa Daurada	0.486
16	Isla de Mallorca	0.741			

Source: Authors' calculations

**Fig. 6:**
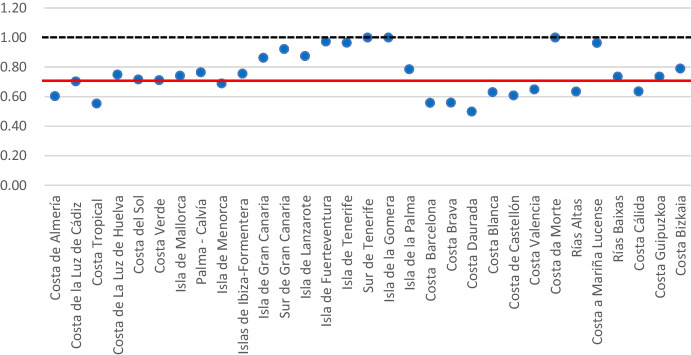
Efficiency scores per tourism zone

Figure 6 presents the efficiency scores for each tourism zone, and the red line therein indicates the average efficiency score, the value of which stands at 0.752. Of the 31 tourism zones studied, only 14 have an above-average efficiency score. The most outstanding areas in terms of efficiency score are located on one of the two mainland archipelagos (Balearic and Canary Islands). Of the 3 efficient areas, 2 are located on islands in the Canary Islands (Isla de la Gomera and Sur de Tenerife) and, in addition, another 8 areas located on islands on the archipelagos obtain an above-average efficiency score. At the opposite extreme are the tourist areas located on the coast of Catalonia (Costa Brava, Costa Barcelona, and Costa Daurada), which obtain very low efficiency scores (Table 4).

These results enable us to state that, although the Spanish tourism market is dominated by mass tourism, the impact of this tourism on employment is unequal, in that it fails to have a significant effect on efficiency in the areas in which it takes place, and very few tourist areas make efficient use of the available resources, thereby confirming one of the research hypotheses.

As a sensitivity and robustness analysis of the DEA model, an alternative DEA model has been considered in which environmental factors have been taken as inputs. The results are shown in Table 5. It can be seen that the results are sensitive to alternative inputs to those initially considered, given that the number of efficient tourist zones increases considerably, from 3 zones in the original DEA model to 13 efficient zones. Furthermore, when considering environmental factors as inputs in the alternative DEA model, the average efficiency score increases considerably, from 0.752 points to 0.913 points. This improvement does not necessarily imply that the alternative model is better, the large number of efficient areas obtained in the alternative DEA model does not seem to reflect the reality of the performance of the tourism areas analysed, while the original DEA model seems to reflect their performance more closely. Since as the existing literature shows, the inputs considered in the original DEA model are more appropriate to measure the objective pursued in the paper, given that this literature considers that employment in the tourism sector is based on the number of tourists, their accommodation needs, and/or the resources on which tourism is related (Deng et al. 2019; Sellers-Rubio and Casado-Díaz 2018; Soysal-Kurt 2017; Huang et al. 2012; Hadad et al. 2012; Lozano and Gutiérrez 2011).

**Table 5:** Efficiency score for the original DEA model and an alternative DEA model.

Tourist area	Efficiency score with DEA alternative inputs	Efficiency score with DEA original inputs
Costa de Almería	1.005	0.603
Costa de la Luz de Cádiz	1.082	0.702
Costa Tropical	0.620	0.553
Costa de La Luz de Huelva	1.256	0.749
Costa del Sol	0.991	0.773
Costa Verde	1.172	0.712
Isla de Mallorca	1.626	0.742
Palma—Calvía	0.853	0.765
Isla de Menorca	0.650	0.689
Islas de Ibiza-Formentera	0.638	0.756
Isla de Gran Canaria	1.078	0.863
Sur de Gran Canaria	0.898	0.923
Isla de Lanzarote	0.875	0.862
Isla de Fuerteventura	0.869	0.973
Isla de Tenerife	1.043	0.965
Sur de Tenerife	1.200	1.134
Isla de la Gomera	0.636	1.251
Isla de la Palma	0.727	0.761
Costa Barcelona	0.287	0.551
Costa Brava	0.696	0.556
Costa Daurada	0.609	0.487
.Costa Blanca	1.069	0.616
Costa de Castellón	1.047	0.600
Costa Valencia	1.076	0.641
Costa da Morte	0.599	1.049
Rías Altas	0.787	0.635
Costa a Mariña Lucense	1.744	0.963
Rías Baixas	1.330	0.736
Costa Cálida	0.729	0.632
Costa Guipuzkoa	0.563	0.736
Costa Bizkaia	0.534	0.791

Source: Authors' calculations

Given the importance of achieving labour efficiency in tourist areas, the next task is to identify the contribution of the inputs to the efficiency results obtained. For this purpose, two groups of tourism zones are distinguished: on the one hand, those with efficiency values above the sample mean, and, on the other hand, those with scores below the mean efficiency score. Table 5 shows the analysis of variance, which leads us to conclude that the null hypothesis of equality of means between the two groups of tourist areas for all inputs is rejected, since the differences in the mean scores of the inputs studied in the two groups are not random. The degree of importance of the inputs in explaining the labour efficiency of the tourist areas is as follows: (1) hotels; (2) overnight stays; (3) beds; and (4) tourists (Table 6). This result is somewhat different from that obtained by Martin et al. (2018), where it is established that the variables that refer to the availability of accommodation and the flow of international visitors are decisive in determining the level of seasonality of the tourist destination. Thus, supply and demand variables are key to characterise the intensity of the tourist flow at certain times of the year. This difference in results is basically due to the fact that Martin et al. (2018) measure tourism seasonality based on indicators that are constructed from individual variables and analyse the amount of information provided by each variable in the construction of the synthetic indicator. Seasonality is precisely the differential element that marks the discrepancies in the results in terms of the discriminating power of the variables.

**Table 6:** Analysis of variance

	Places	Hotels	Tourists	Overnight stays
Mean of group A	40,078.790	129.280	1,871,047.210	10,791,028.790
Mean of group B	31,941.400	227.030	1,990,947.650	7,755,352.820
Statistic F	4.123	4.864	3.195	4.570
P-value (p)	0.049*	0.038*	0.061**	0.041*

Group A: efficiency index greater than mean for the whole sample: 14 tourist areas

Group B: efficiency index smaller than mean for the whole sample: 17 tourist areas

*p < 0.05 and **p < 0.1

Source: Authors' calculations

### Analysis of environmental factors

The analysis of the results obtained for the efficient tourist areas suggests that there may be a relationship between labour efficiency and the location of the tourist area, given that the tourist areas with the highest efficiency are those located on the peninsular archipelagos. Therefore, the evaluation of the location of the tourist destination as a factor that has a significant influence on efficiency is considered, whereby the possible relationship between the measurement of efficiency in the input and the location of the tourist area is studied. This involves determining whether or not there are significant differences in the mean values of in the efficiency scores between the two groups in terms of whether the tourist area is located on the mainland or on an archipelago. The Analysis of Variance methodology (Statistic F = 12.869; p = 0.001) leads to the rejection, with a 1% level of significance, of the hypothesis of equality of means for the input efficiency scores in the two groups of tourist areas according to their location. When studying the mean efficiency values for each group (Table 7), it can be observed that the tourist areas located on certain islands of the archipelagos are more efficient on average than the tourist areas located on the mainland. These results confirm the positive effect of the location of the tourist destination on labour efficiency.

**Table 7:** Average efficiency scores by location of the tourist destination

	Archipelago	Peninsula	Global
Mean	0.890	0.688	0.752
S.D	0.162	0.136	0.177

Source: Authors' calculations

In order to continue with the study of environmental factors, it is also analysed whether variables other than those used in the DEA model influence efficiency. It must be considered that the inclusion of too many variables in the DEA model can reduce theidentification capacity of the model (Lee et al. 2012). For this reason, a Tobit-type regression model is used (Coelli et al. 1998; Perrigot and Barros 2008; De Jorge and Suárez 2014).

Three variables are considered that were not selected as inputs or outputs in order not to reduce the explanatory power of the DEA model (Lee et al. 2012). Two of these variables are related to the market: the length of stay (Length of stay), and the number of international tourists (Foreigners). On the other hand, a variable related to the tourist destination is considered, which measures the quality of the destination through the hotels awarded a Q (Quality) quality mention.

Recall that the specified model is as follows:

$$E_j=\alpha_0+\delta_1{Foreigners}_j+\delta_2{Length\;of\;stay}_j+\delta_3{Quality}_j+\varepsilon_j,\;j=1,\;\dots,31$$

where E is the efficiency score, α _0_, ${\delta }_{1}$, ${\delta }_{2}$, ${\delta }_{3}$ are the parameters to be estimated, and ε is a white-noise variable. In this model, the dependent variable (efficiency score) represents the inefficiency mode, and therefore an estimated parameter with a negative sign indicates a positive effect on efficiency, while an estimate with a positive sign indicates a negative effect on efficiency.

With regard to the significance of the variables, all the variables under study were found to be statistically significant (Table 8). With respect to the working hypotheses, it is confirmed that the influence of length of stay, number of international tourists, and tourism quality all exert a positive impact on labour efficiency. The fact that length of stay has a positive effect on efficiency shows that destinations and tourism establishments must work together to offer a significant variety of activities to encourage and motivate tourists to extend their length of stay. To a certain extent, this variable captures the capacity of the tourist area to attract new visitors. The number of foreign tourist arrivals has a positive effect on efficiency, which indicates that the high purchasing power and larger travel budget of this type of tourist exert a greater impact on the labour market, thereby highlighting the importance of the foreign tourism market. The positive effect of the tourist quality of hotels on efficiency shows that greater inputs are needed to offer a high-quality service. Quality is a major determinant in attracting high-income tourists, staying in higher-quality hotels, and paying higher rates, which means that the hotel may need more labour to maintain the required quality standard and, therefore, the final effect on efficiency is positive. This influences the hotel's commitment to implement a quality system, which entails major investment.

**Table 8:** Estimation and validation of the Tobit model for the analysis of environmental factors

Variables	Estimated parameters	P-value (p)
Constant	1.820	0.180
Foreigners	-1.940 × 10^–07^	0.049*
Length of stay	-0.258	0.031*
Quality	-0.059	0.036*
σ_ε_	0.743	0.000**
R^2^	0.499	
R^2^ adjusted	0.422	
Log likelihood	-16.261	

*p < 0.05 and **p < 0.01. Source: Authors' calculations

#### Robustness analysis

The presence of a potentially endogenous explanatory variable (understood as the existence of correlation between this variable and the error term) in the equation that modelling the environmental factors that determine efficiency can be interpreted as a problem of simultaneity or the existence of omitted or unobservable variables. In such a case, estimates that include the potentially endogenous variable in the equation under study as an additional exogenous variable are biased. The endogeneity test (Hausman test) is applied to perform this analysis, where the relationship between the explanatory (independent) variables and the disturbance variable is studied. Under the null hypothesis, the model is correctly specified with all explanatory variables as exogenous. Under the alternative hypothesis, the residuals from a linear regression of the potentially endogenous variable on a set of instruments are included in the regression. Rejection of the null hypothesis would imply that these residuals are significant and therefore there would be an endogeneity problem, which would imply the estimation of an alternative model to the one initially specified. The endogeneity test with the variables Foreigners, Length of stay Quality has shown that they are not endogeneous (the p-values are 0.157, 0.094 and 0.254 respectively), so the model proposed with the selected environmental factors is correct. On the other hand, the variance inflation factor is calculated by auxiliary regression to diagnose the multicollinearity of the processed independent variables. This test gives a value of VIF = 3.142, which is less than 10, so according to the empirical principle, there is no multicollinearity between the independent variables.In order to test the Tobit regression variables and ensure the robustness of the model, the Least Squares and Fully Modified Least Squares models are estimated, thereby testing the impact of external structural factors on efficiency (Faleye et al. 2014; Li et al. 2020). For the different regression models estimated (see Table 9), the significance test results are consistent with the Tobit regression results, showing that the specified Tobit model is robust.

**Table 9:** Robustness Analysis

Method: Least Squares		
Variables	Estimated parameters	P-value (p)
Constant	0.010	0.969
Foreigners	-6.630 × 10^–09^	0.045*
Length of stay	-0.107	0.047*
Quality	-0.003	0.045*
R^2^	0.411	
R^2^ adjusted	0.346	
Method: Fully Modified Least Squares		
Variables	Estimated parameters	P-value (p)
Constant	0.067	0.814
Foreigners	-5.610 × 10^–09^	0.036*
Length of stay	-0. 113	0.046*
Quality	-0.004	0.050*
R^2^	0.397	
R^2^ adjusted	0.328	

*p < 0.05

Source: Authors' calculations

Finally, the stability study of the model is carried out for the different groups of tourist areas reflected in the analysis. For this analysis, we first studied the stability of the model when considering the grouping of the tourist areas according to whether the efficiency is higher or lower than the average, in which case the Chow test, at a 5% significance level (F-statistic = 1.776, p-value = 0.183), shows that the model is stable for the sub-samples considered, with no structural change. In the case of considering the grouping corresponding to the location (archipelago or peninsula), the Chow test (F-statistic = 1.052, p-value = 0.351) also shows the stability of the model.

## Discussion and conclusions

This paper studies mass tourism, which is usually associated with so-called sun-and-sand tourism. The study is motivated by the importance and weight of tourism in the Spanish economy, since Spain constitutes the world leader in holiday tourism destinations. This work contributes to the literature on tourism efficiency, and proposes an innovative model of geographical analysis, whose main contribution involves the territorial units studied. Its analysis is centred on coastal tourist areas: these destinations are smaller geographical spaces than those hitherto examined in the literature on efficiency. The analysis employs stochastic DEA applied to hospitality industry data set in 31 Spanish tourist areas for the year 2019. The role of four environmental variables on labour efficiency is also examined.

The analysis of the tourist areas located on the Spanish coastline shows that the impact of mass tourism on labour efficiency is uneven, with a high degree of labour inefficiency for the areas examined. The challenge is to manage the tourism sector, achieving efficiency with the resources available (Blanke and Chiesa 2008). This is especially important in countries such as Spain, where tourism is an important factor for development. To help in this challenge, it is important to know the most relevant inputs that explain the labour efficiency of tourist areas, these being in order of importance: the number of hotels, number of overnight stays, number of bedplaces and number of tourists. This result shows the importance of tourist resources as a lure for sun and sand tourism in Spain. This result is somewhat different from other research which shows that the impact of tourism depends to a large extent on two characteristics of the activity developed, such as the total number of arrivals and the degree of concentration of tourists at certain times of the year (Martín et al. 2017, 2018). It can be said that the predominance of the sun and sand model present in Spain is not positively associated with labour efficiency, which shows that the net effect on the labour efficiency of the tourism sector in Spanish coastal areas is insignificant.

It has been found that there are important geographical differences that make it impossible to ignore the spatial perspective (Sellers-Rubio and Casado-Díaz 2018; Solana-Ibáñez et al. 2016), highlighting the complexity of the level of efficiency of the Spanish labour market. The location of tourist areas is a determining factor for efficiency, with the peninsular archipelagos of the Balearic and Canary Islands achieving the highest degree of efficiency. There is a labour imbalance that favours the tourist areas located in some of the islands that make up the archipelagos. These areas are very important sun and sand tourist markets for the country, where international tourism plays a fundamental role (Cordero and Tzeremes 2017). The location of the archipelagos gives them a competitive advantage over other destinations (Martín et al. 2018), showing that the territorial framework is especially relevant when the information analysed refers to administrative units that are not necessarily traditional labour market areas (Sánchez-Sánchez et al. 2021; Sánchez and Sánchez 2018).

The results obtained have important implications from the territorial perspective studied, the benchmarking process requires identifying the underlying causes of territorial differences. In terms of efficiency, this implies that inefficient tourist areas should examine the reasons why other areas are more efficient. Thus, the efficiency with which different tourist areas operate allows the identification of the determinants of their different levels of efficiency, which allows the value of the different strategies adopted to be measured. In particular, the results show a significant and positive impact on the efficiency of the factors number of international tourists (Hwang and Chang 2003; Chen 2010; Huang et al. 2012; Ben Aissa and Goaied 2016; Assaf et al. 2017; Sellers-Rubio and Casado-Díaz 2018), length of stay (Parte-Esteban and Alberca-Oliver 2015; Sellers-Rubio and Casado-Díaz 2018) and hotels with quality distinctions (Costa 2004; Claver-Cortés et al. 2007; Arbeló-Pérez et al. 2017), which highlights the importance of developing marketing strategies focused on the international tourism market, offering activities to increase the length of stay in the tourist destination and promoting commitment to quality to act as a tourist attraction. These results coincide with some studies focused on mass tourism in Spain (Baidal et al. 2013; Claver et al. 2007). The study developed provides a starting point for the analysis of factors that cause the observed differences in efficiency. The lack of information in the territorial units considered prevents the analysis of other determinants of efficiency, such as, for example, investment in advertising campaigns, the media used for marketing campaigns, the strength of the destination brand, the image of the tourist destination, etc.

The results obtained provide useful information to tourism managers, who must formulate strategies and promote employment policies with the aim of contributing to labour efficiency, improving productivity and assisting in the decision-making process of tourism managers. In Spain, institutions carry out important marketing campaigns with the aim of promoting tourist destinations and attracting new tourists, and the efficiency estimates can be taken as external benchmarks.

For future research, it would be interesting to validate the model proposed here in other countries where the demand for sun and sand tourism is similar to that of Spain, given that they are direct competitors of the country. Such a model can be extended by considering other territorial units of analysis, which could be smaller geographical units such as municipalities, cities, tourist spots, etc., in line with research such as that of Marco-Lajara et al. (2016) where hotel performance in tourist districts is analysed. It is also possible to consider the effect of the COVID-19 pandemic on the model proposed, given that the special circumstances posed by the health crisis that began in 2020, may cause significant effects on the efficiency of tourist destinations. Particularly interesting is the analysis of the determinants of performance, where factors such as the incidence rate of COVID-19, health restrictions (confinement of people, border closures or local perimeter closures), the security measures adopted by hotels or the perception of risk about the tourist destination, may have played an important role on the image of the tourist destination, affecting tourism in some areas, which may have determined the performance of tourism.

## Footnotes

1 Censored sample is a sample in w hich information on the regressand is available only for some observations but not for all, although we may have information on the regressors for all the units in the sample. Further, we may have the case of runcated sample model, in which information on both the regressand and regressors is not available on some observations (see Wooldridge 2002).
